# Allogeneic Transplant in ELANE and MEFV Mutation Positive Severe Cyclic Neutropenia: Review of Prognostic Factors for Secondary Severe Events

**DOI:** 10.1155/2017/5375793

**Published:** 2017-01-18

**Authors:** Onyemaechi N. Okolo, Emmanuel Katsanis, Seongseok Yun, Candace Y. Reveles, Faiz Anwer

**Affiliations:** ^1^Department of Medicine, University of Arizona, Tucson, AZ, USA; ^2^Department of Pediatrics, University of Arizona, Tucson, AZ, USA; ^3^Division of Blood & Marrow Transplantation, University of Arizona, Tucson, AZ, USA; ^4^Department of Pathology, University of Arizona, Tucson, AZ, USA; ^5^Division of Hematology and Oncology, University of Arizona, Tucson, AZ, USA

## Abstract

*Objective and Importance.* Cyclic neutropenia (CyN) is a rare autosomal dominant inherited disorder due to the mutation ELANE primarily affecting bone marrow stem cells and is characterized by recurrent neutropenia every 2 to 4 weeks. Symptoms vary from benign to severe, including death. Postulations on the cause of wide spectrum in symptom presentation include the possibility of other genetic mutations, such as MEFV. Recommended treatment for CyN is G-CSF to keep ANC higher to minimize risk of infection.* Case.* A 25-year-old male diagnosed with CyN, on G-CSF but worsening quality of life. Pretransplant investigations revealed ELANE mutation positive severe CyN along with familial Mediterranean fever (MEFV) mutation.* Intervention.* Bone marrow transplantation as treatment for dual mutation (ELANE and MEFV mutation) positive severe CyN.* Conclusion.* BMT may be considered as an alternative treatment for severe CyN in patients who are refractory to G-CSF. It is postulated that in our patient the combined mutations (CyN and MEFV) may have contributed to the severity of this individual's symptoms. We suggest CyN patients who present with severe symptoms have evaluation with ELANE mutation testing, Periodic Fever Syndromes Panel, and routine marrow assessment with FISH, conventional cytogenetics, and morphological evaluation for MDS/AML.

## 1. Introduction

Cyclic neutropenia (CyN) is a rare stem cell disorder with a prevalence of one to two per million [1]. It results from a heterozygous mutation in the ELANE (full length) gene that encodes neutrophil elastase on chromosome 19p13.3 [2]. Horwitz et al. hypothesized that the cellular mechanism in CyN is likely due to gain-of-function mutation and proteolysis [2]. The abnormal enzyme resulting from mutated ELANE gene damages hematopoietic cells as they differentiate to the neutrophil lineage [3]. The damage occurs through the initiation of the unfolded protein response, which accelerates apoptosis of developing myeloid cells [4].

Three to five days of profound neutropenia (<0.2 × 10^9^/L) recurs every 21 days in more than 90% of CyN patients, although the cycle can range from 2 to 4 weeks [5]. The manifestations may be variable and include fever, lymphadenopathy, mouth ulcers, and infections such as sinusitis, pharyngitis, cellulitis, pneumonia, and acute peritonitis. Symptoms are usually recurrent, and sometimes severe, having led to death [5]. The standard of care for CyN is the use of granulocyte colony stimulating factor (G-CSF), which leads to an increase in absolute neutrophils counts and reduces neutropenic periods and severe events [6]. Although not common in CyN, myelodysplastic syndrome (MDS) or acute myeloid leukemia (AML) transformation is a well-known complication observed in cases of severe congenital neutropenia (SCN), a similar but noncyclical neutropenia caused by mutation of the ELANE gene, and less frequently, the GFI1 gene [7]. Transformation has been shown to occur in SCN that has been treated with G-CSF (seen in 16% of patient studied by Makaryan et al.) [4]. There is limited literature on utilizing allogeneic bone marrow transplant (BMT) to treat SCN [6], as it is rarely used for treatment of only very severe conditions of neutropenia.

Familial Mediterranean fever (FMF) is a hereditary autoinflammatory disorder caused by a mutation of the MEFV (Mediterranean fever) gene, which is responsible for making pyrin. A missense mutation or a deletion of the gene leads to dysfunction of pyrin [8]. This protein is present in neutrophils and we report the first case of cyclic neutropenia with ELANE mutation in combination with MEFV mutation resulting in severe symptoms. We hypothesize that this unique combination of mutations in our patient may have contributed to severe symptoms resulting in need for allogeneic stem cell transplantation.

## 2. Case Presentation

The patient is a 25-year-old Caucasian male with CyN, who was diagnosed at age one. Since childhood he suffered through multiple hospitalizations for severe infections and required numerous surgeries related to his underlying neutropenia. He had an extensive history of oral mucosa lesions, throat, ear, fingernail and buttock infections, and bouts of bloody diarrhea with colitis. His surgeries included tonsillectomy, mastoidectomy, appendectomy, and cholecystectomy. His quality of life had been deteriorating, and the patient voiced unhappiness. His neutropenic cycles were initially every 25–28 days with 3–5 days of nadir, and he had temporary improvement in his cycles with filgrastim treatment. Neutropenic cycles shortened to 15–18 days but he remained vulnerable to infections during his nadirs and suffered opportunistic infections on a number of occasions despite G-CSF, which was more recently replaced with PEGylated G-CSF.

**Figure 1:**
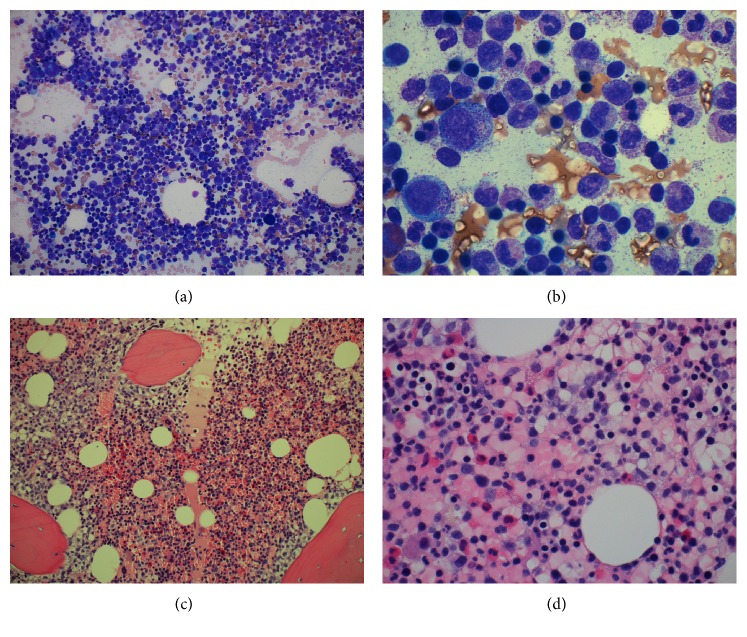
Myeloid hyperplasia with prominent granulation and a shift to immaturity consistent with growth factor (G-CSF) effect. (a) Bone marrow aspirate low power (20x), (b) bone marrow aspirate high power (100x), (c) bone marrow core biopsy (20x), and (d) bone marrow core biopsy (60x).

Following evaluation of the patient at our institution, he underwent testing for ELANE mutation, which identified c.573_597 + 5del, heterozygous, frame-shift deletion mutation of neutrophil elastase, located in the neutrophils. Additionally, the Periodic Fever Syndromes Panel was performed (testing for LPIN2, MEFV, MVK, NLRP3, PSTPTP1, and TNFRSF1A) and returned positive for MEFV (Mediterranean fever) with nucleic acid change c. [1105C>T; 1223G>A] and amino acid alteration p. [Pro369Ser; Arg408Gln]. Bone marrow biopsy (core and aspirate; Figures 1(a), 1(b), 1(c), and 1(d)) showed myeloid hyperplasia with prominent toxic granulation and a shift with immaturity consistent with G-CSF effect, but with no overt dysplasia and only 1% blasts, and otherwise normal hematopoiesis with no evidence of malignancy.

**Figure 2:**
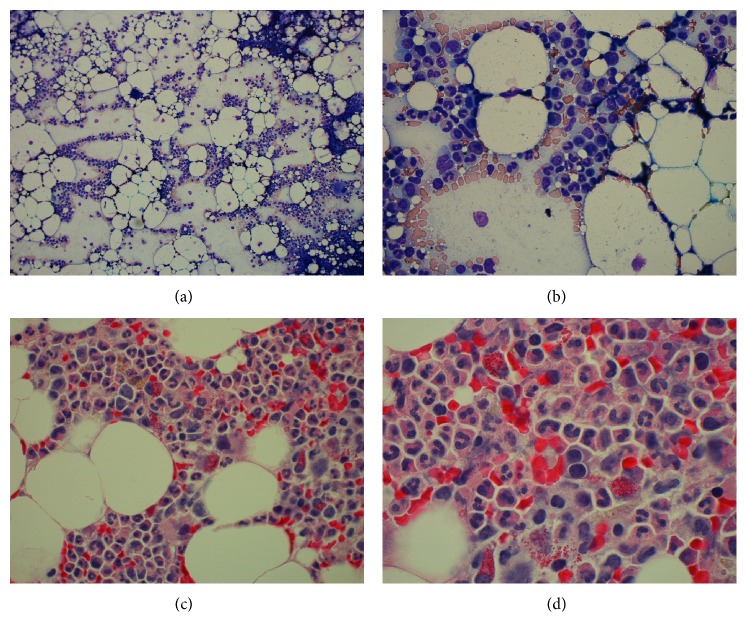
Engrafting bone marrow with trilineage hematopoiesis and decreased cellularity compared to previous marrow (30% versus 80%). (a) Bone marrow aspirate low power (10x), (b) bone marrow aspirate high power (60x), (c) bone marrow core biopsy (40x), and (d) bone marrow core biopsy (100x).

Given the G-CSF refractory neutropenia and progressive symptomatic disease, the decision was made to proceed to off protocol, institutional standard allogeneic BMT. In the absence of matched sibling, patient received a 10 of 10 antigen matched unrelated bone marrow hematopoietic stem cell transplant. The patient was given conditioning chemotherapy using combination cyclophosphamide (Cy), fludarabine (FLU), anti-thymocyte globulin (ATG), and low dose total body irradiation (200 cGy). The patient was started on methotrexate (10 mg/m^2^ on days +1, +3, +6, and +11) and cyclosporine (120 to 150 mg BID dose and goal trough level 200–250) for GvHD prophylaxis. Posttransplant course was complicated by neutropenic fever and signs of engraftment syndrome with mild tachypnea and generalized rash; this responded to a short-term course of prednisone. By day +14, the patient engrafted for neutrophils and platelets (Table 1), and bone marrow biopsy (core and aspirate; Figures 2(a), 2(b), 2(c), and 2(d)) and DNA test at 2 months confirmed 100% donor chimerism. He was discharged from hospital on day +20 with no evidence of GvHD, feeling well and eating normally. He was seen in clinic initially weekly and then every two weeks for his normal follow-up appointments and he remained well and asymptomatic. He presented on day +53 after BMT with a 7-day history of severe diarrhea and was admitted to the hospital. Endoscopy showed granularity and cobble stone appearance in the small bowel, consistent with GI GvHD. Biopsy obtained from endoscopy revealed grade 4 of 4 GI GvHD, with extensive mucosal ulceration and development of pneumatosis cystoids intestinalis, acute serositis, and likely focal microperforation. Despite treatment with steroids, calcineurin inhibitors, cellcept, budesonide, and infliximab, the grade IV GI GvHD progressed and he developed perforation of the cecum requiring subtotal colectomy. After a prolonged hospital stay, patient died of respiratory failure and septic shock +128 days following BMT.

## 3. Discussion

Cyclic neutropenia is a hematopoietic stem cell disorder, which was demonstrated by Krance et al. after the unintended transfer of the disorder from an affected (but generally asymptomatic) individual to her unaffected sister via bone marrow transplant in order to treat acute lymphoblastic leukemia (ALL) [18]. Phenotypic presentation of the disease is variable, but a correlation between mutation type and disease severity may exist, which may help predict the patient population that would benefit from more aggressive therapy. Lange and Jones compiled a list of deaths related to CyN, which showed that many patients died due to sepsis, colitis, pulmonary complication, lymphosarcoma, and massive gastrointestinal hemorrhage [19]. Few neutropenia patients who are refractory to routine therapy and have severe symptoms undergo treatment with allogenic stem cell transplantation [7], but there is paucity of published literature on this subject [8, 20, 21] (see Table 2 for summary).

Stem cell transplantation has been used to treat congenital neutropenia that is unresponsive or only partially responsive to G-CSF since the 1970s [20]. A report on 11 patients (age 6 months to 15.9 years) between 1976 and 1998 who underwent transplant is one of the first truly comprehensive works that provided information regarding long term outcomes—in fact one patient was followed up for 22 years after transplant at the time of publication [20]. The report showed that all patients who received HSCT from a sibling had survived, one patient had severe GvHD, and all but one patient continued to require G-CSF secondary to transplant rejection [20]. Other studies have shown that if a sibling donor is not available, an unrelated HLA-matched BMT can be successful even in situation of pretransplant infection [21]. A general study of patients with inherited immunodeficiency states was conducted by Amrolia et al. and they reported that nonmyeloablative SCT allowed for fast engraftment from both related (sibling) and unrelated matched donors with minimal toxicity (conditioning regimen: fludarabine-melphalan-antilymphocyte globulin) [22].

Our patient had a history of multiple hospitalizations due to recurrent fevers and infections including perirectal abscess as well as intermittent bloody diarrhea from colitis, which were of major concern. His phenotypic presentation was high risk, though the risk associated with his specific ELANE mutation (c.573_597+5del, heterozygous, frame-shift deletion mutation) is unclear. According to Germeshausen et al., there is, as of yet, no clear genotype-phenotype correlation with ELANE mutations and predicted severity of disease, suggesting that a wide range of other mechanisms including epigenetic and environmental factors may play an important role as well [23]. In an attempt to assess the correlation of specific mutations and disease severity, Makaryan et al. showed that the cumulative incidence of a severe event (defined as MDS/AML transformation, transplant, or death) in SCN and CyN patients after 20 years of G-CSF treatment was over 70% if the mutation was located in exon 5, over 50% if the mutation was located from the 5′ UTR region through exon 2, and 35% for patients whose mutation was located in the interior of the gene from exon 3 through intron 4. Moreover, those with mutations G214R and C151Y all experienced a severe event, 10% with the S126L mutation had a severe event, and none with P139L or IVS4+5G>A had a severe event, and of the individuals studied, P139L, IVS4+5G>A, and S126L were seen in both SCN and CyN whereas C151Y and G214R were seen only in SCN [4]. The study showed that, after 20 years of G-CSF, the risk of a severe event in SCN was 46% and 7% in CyN. Further, they found that the ELANE genotype may influence the risk of severe bacterial infections and that the dose of G-CSF needed to treat neutropenia also correlated to mutation type [4]. Beekman and Touw state that secondary leukemia in SCN likely arises because of chronic genotoxic stress in the HSC compartment, which leads to acquisition of oncogenic mutations [24].

G-CSF is considered standard treatment of CyN [6] and although our patient had initially benefitted from G-CSF, during later years he experienced multiple severe infections. Moreover, malignant transformation to MDS or AML has been reported as one of the most serious complications of prolonged G-CSF treatment [24]. One study with SCN patients by Beekman and Touw demonstrated MDS/AML risk to increase over time from 2.9% per year after 6 years to 8% after 12 years [24], and individuals that required more than the median dose (8 mcg/kg/day) were shown to have incidence up to 40% after 12 years compared to 11% in patients who responded to lower doses. MDS/AML transformation upon long term G-CFS use has not been reported in CyN patients [24], although it is also possible that these findings of transformation in SCN may be due to longer duration with disease that transforms to malignancy on its own.

Currently, no standard treatment algorithm exists to determine when a patient with CyN should be considered for allogeneic BMT when refractory to G-CSF. Allogeneic BMT is currently the only alternative therapy for those with SCN who are refractory to G-CSF [7, 10, 12] but there is limited data on outcomes. Fioredda et al. report outcomes on 136 SCN patients who underwent BMT. Their data showed a 3-year-overall survival posttransplant of 82% and transplant related mortality of 17% [10]. Younger patients (less than 10 years of age at time of transplant) did better overall [10]. There was a 21% cumulative incidence of GvHD at day +90 and 20% cumulative incidence at 1 year [10]. Another study by Oshima et al. reported a cumulative incidence of grade II-IV GvHD of 11% [12]. The difference between both studies may be due to median age of subjects (younger in the Oshima group) or type of conditioning regimen utilized. Due to the similarities between CyN and SCN, allogeneic BMT is a potentially curative treatment for individuals only with severe CyN. Transplant use may be considered as the last measure for patients due to its high risks associated with transplantation, including GvHD and transplant related mortality. In SCN, it is critical to quickly detect signs of malignant transformation in order to pursue alternatives treatment such as allogeneic BMT in a timely manner due to risk of transformation to MDS/leukemia [7]. Although watchful waiting is considered an acceptable option, it is important to keep in mind that, in cases of SCN, success rates of transplantation in more advanced stages of malignant transformation are less than earlier stage [23], and an earlier age of transplantation has been shown to lead to long term success [7], indicating the need for systematic approach to decide optimal time for transition to BMT from G-SCF.

In addition to ELANE mutation, our patient had positive finding of familial Mediterranean fever (MEFV). MEFV is an autosomal recessive disorder characterized by mutation of the gene MEFV that produces the protein pyrin found within cytoskeletons of certain WBCs including neutrophils [25]. Characteristics of this mutation include recurrent but self-limited episodes of fever, pleuritis, arthritis, and peritonitis [25]. This unique dual mutation finding in our patient may have contributed to his severe symptoms due to higher susceptibility to infections, although this cannot be confirmed because the specific amino acid alteration of MEFV found in our patient can present with typical familial Mediterranean fever which can have an asymptomatic presentation [26].

## 4. Conclusion

Cyclic neutropenia has a wide range of phenotypic presentations. Treatment with G-CSF is adequate for many affected by CyN but few are refractory to treatment with G-CSF. Allogenic transplantation is option of last resort but it is potentially curative. Secondary severe event (MDS/AML transformation, transplant, or death) in SCN and CyN patients after 20 years of G-CSF treatment is seen over 70% if the ELANE mutation was located in exon 5, over 50% if the mutation was located from the 5′ UTR region through exon 2, and 35% for patients whose mutation was located in the interior of the gene from exon 3 through intron 4. Mutations G214R and C151Y are linked severe event. Only 10% with the S126L mutation had a severe event and P139L or IVS4+5G>A is not linked with severe event. After 20 years of G-CSF, the risk of a severe event in SCN is 46% and 7% in CyN. Other than factors described above, there is no other correlate for CyN genotype to phenotype in order to predict severity. We suggest patients who present to transplant physicians for evaluation need to have ELANE testing along with additional comprehensive testing like Periodic Fever Syndromes Panel testing for LPIN2, MEFV, MVK, NLRP3, PSTPTP1, and TNFRSF1A, in addition to routine marrow assessment with FISH and conventional cytogenetics and morphological evaluation for MDS/AML. This first report suggests that combined ELANE/MEFV mutations may have worse prognosis due to severe symptoms in CyN.

## Figures and Tables

**Table 1:** Laboratory results before and after allogeneic BMT.

	Day −5	Day −2	Day +7	Day +27	Day +41	Day +78
WBC (1000/uL)	18.5	4.9	<0.1	2.9	2.9	5.7
ANC (1000/uL)	16.3	4.90	Too few	1.50	1.80	5.50
Hemoglobin (g/dL)	11.7	10.8	6.5	10.6	7.5	11.8
Platelet (1000/uL)	140	56	31	227	164	77

**Table 2:** Summary of relevant literature of SCN and HSCT.

Article reference	Subjects	Demographics	Treatment	Conclusion
[9]	1	4 y/o male with SCN	HSCT: matched unrelated donor (MUD)	“HSCT is a useful treatment for SCN patients, especially those who are at high risk for leukemic transformation”

[10]	136	0–43 y/o male and females with SCN	HSCT (i) 61 HLA matched related donors (ii) 61 HLA-MUD (iii) 14 mismatched donors	“3-year overall survival (OS) was 82%, and transplant-related mortality (TRM) was 17%… Cumulative incidence (1 year) of chronic GVHD was 20%”

[11]	7 (one subject transplanted twice)	2.8–28 y/o males and females with SCN	HSCT (i) 3 HLA matched siblings (ii) 3 HLA-MUD (iii) 2 cord blood	“Two of seven (29%) patients died; both had MDS/L… one patient has chronic GVHD 2 years post-transplant”

[12]	18	0.2–16.7 y/o males and females with SCN	HSCT (i) 9 HLA matched siblings (ii) 9 HLA-MUD	“Engraftment occurred at the first HSCT in 12 patients, four patients received a second HSCT for graft failure, and two patients died. The cause of death was renal failure and graft failure at the first and second HSCT, respectively. The cumulative incidence of grade II–IV acute GVHD and TRM at the first transplantation was 11% and 5.6%, respectively”

[13]	N/a. Review of guidelines and treatments	Males and females with SCN, leukocyte adhesion deficiency, and chronic granulomatous disease	HSCT with matched and medically unrelated donors	“Allogeneic stem cell transplantation and, possibly, gene-replacement therapy are the only curative treatments available”

[14]	Review of 300 patients on Severe Chronic Neutropenia International Registry (SCNIR)	Males and females with SCN	(i) GCSF (ii) HSCT	“More than 90% of patients respond to recombinant human (rHu) G-CSF with ANCs that can be maintained at approximately 1.0 × 10(9)/L… Hematopoietic stem cell transplantation (HSCT) is still the only available treatment for patients refractory to rHuG-CSF treatment”

[15]	600 patients with CN collected by the SCNIR	Males and females with SCN	(i) GCSF (ii) HSCT	“In recent analyses the influence of the G-CSF dose required to achieve neutrophil response (ANC > 1,000/microL) in the risk of developing acute myeloid leukemia (AML) has been reported”

[7]	101 SCN, 9 of which received HSCT	Males and females with SCN	HSCT (i) 2 HLA matched related donors (ii) 7 HLA-MUD	“HSCT is feasible for patients with SCN who do not respond to G-CSF, who have malignant transformation, or who are at a high risk of malignant transformation, even if an HLA-identical sibling donor is not available”

[16]	300 patients from SCNIR	Males and females with SCN	(i) GCSF (ii) HSCT	“Adverse events documented in this group of patients include splenomegaly, thrombocytopenia, osteoporosis and malignant transformation into MDS/leukemia. If and how rHuG-CSF treatment impacts on these adverse events remains unclear since there are no historical controls for comparison. For those patients who are refractory to rHuG-CSF treatment and continue to have severe and often life-threatening bacterial infections, hematopoietic stem cell transplantation (HSCT) is still the only currently available treatment”

[17]	N/a (review of characteristics, diagnosis, management, and genetic counseling)	Males and females with SCN	GCSF (i) HSCT	“Treatment with granulocyte colony-stimulating factor (G-CSF) ameliorates symptoms and reduces infections in almost all affected individuals. For affected individuals with a well-matched donor, hematopoietic stem cell transplantation (HSCT) may be the preferred treatment option. HSCT is the only alternative therapy for individuals with congenital neutropenia who are refractory to high-dose G-CSF or who undergo malignant transformation”
